# Apixaban for oral antithrombotic therapy: is a new era coming?

**DOI:** 10.1186/2052-8426-2-4

**Published:** 2014-02-01

**Authors:** Yong Zhang

**Affiliations:** Pulmonary Department, Fudan University Zhongshan Hospital, Shanghai, 200032 China

**Keywords:** Apixaban, Factor Xa inhibitor, Antithrombotic therapy

## Abstract

Apixaban, a new oral inhibitor of activated factor Xa, may simplify antithrombotic therapy with fixed doses and no necessity for coagulation monitoring. Apixaban is non-inferior to conventional therapy (enxoaparin, followed by warfarin) with lower risk of major bleeding in the treatment of acute venous thromboembolism. Compared with placebo, extended treatment with apixaban may reduce the recurrence rate in patients with venous thromboembolism. Thromboprophylaxis therapy with apixaban in surgery (hip or knee replacement) and atrial fibrillation has been proved to be superior to warfarin or enxoaparin. Apixaban offers a convenient and more effective alternative choice in anticoagulant therapy.

## Background

Warfarin, as an efficient anticoagulant drug, has been in clinical use for about 60 years. However, many food and drug interactions increase the risk of bleeding and this, in combination with the need for frequent monitoring, makes it a burdensome therapy. Enoxaparin, another anticoagulant drug, can only be administered subcutaneously which is an inconvenient route. Apixaban, a new oral inhibitor of activated factor Xa may simplify antithrombotic therapy with fixed doses and no requirement for of coagulation monitoring. There have been recent clinical trials focusing on the efficacy and safety of apixaban in the treatment and prophylaxis of thromboembolism. Apixaban is safe and effective if it is used appropriately.

## Discussion

A recent large sample trial reported the efficacy and safety of apixaban versus conventional therapy (subcutaneous enxoaparin, followed by warfarin) in symptomatic proximal deep-vein thrombosis or pulmonary embolism [1]. In this double-blind study, 5395 patients were randomized into an apixaban (at a dose of 10 mg twice daily for 7 days, followed by 5 mg twice daily for 6 months) group and a conventional therapy (subcutaneous enoxaparin, followed by warfarin for 6 months) group. Symptomatic recurrent venous thromboembolism or death related to venous thromboembolism occurred in 2.3% of patients who received apixaban and in 2.7% who received warfarin. Major bleeding occurred in 0.6% of patients in the apixaban group, compared with 1.8% of patients who received conventional (P < 0.001 for superiority). Apixaban was non-inferior to conventional therapy with lower risk of major bleeding.

Another recent study indicated that extended low dose apixaban treatment may reduce the recurrence rate in the venous thromboembolism patients who had completed 6 to 12 months of anticoagulation therapy [2]. During the 12 month therapy period, symptomatic recurrent venous thromboembolism or death from venous thromboembolism occurred in 73 of the 829 patients (8.8%) who were receiving placebo, as compared with 14 of the 840 patients (1.7%) who received 2.5 mg of apixaban and 14 of the 813 patients (1.7%) who received 5 mg of apixaban (P < 0.001 for both comparisons). The rates of major or clinically relevant non-major bleeding were similar in the placebo group (2.8%) and 2.5 mg apixaban group (3.2%).

Apixaban may also effectively prevent thromboembolism. In two randomised double-blind trails, when compared with enoxaparin 40 mg daily, apixaban 2.5 mg twice daily administration was associated with lower rates of venous thromboembolism after knee and hip replacement (P < 0.001 and 0.0001, respectively) without increased bleeding [3, 4]. Apixaban offers a convenient and more effective alternative to enoxaparin of thromboprophylaxis in surgery. In patients with atrial fibrillation, apixaban at a dose of 5 mg twice daily was also superior to warfarin in preventing stroke or systemic embolism, it caused less bleeding and resulted in lower mortality (P < 0.001) [5].

However,in some circumstances,apixaban was still associated with increased bleeding risk. With acutely ill patients, with at least one additional risk factor for venous thromboembolism, the long-term prophylactic use of apixaban, administered orally at a dose of 2.5 mg twice daily for 30 days was not superior to a shorter course with enoxaparin subcutaneously at a dose of 40 mg once daily for 6 to 14 days. Apixaban was associated with significantly more major bleeding events than enoxaparin [6]. The addition of apixaban orally, at a dose of 5 mg twice daily, to antiplatelet therapy in high-risk patients after suffering acute coronary syndrome increased the number of major bleeding events without a significant reduction in recurrent ischemic events [7].

## Conclusion

**Figure 1:**
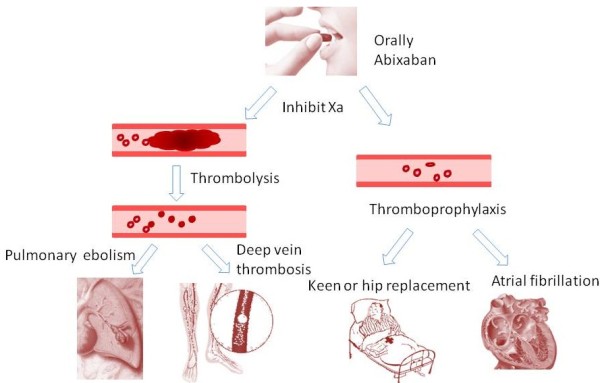
**Clinical application of apixaban.** The application of apixaban in the treatment of venous thromboembolism patients and thromboprophylaxis in surgery and atrial fibrillation patients.

Apixaban, the oral factor Xa inhibitors offers a legitimate alternative to warfarin and enoxaparin. This novel, breakthrough, oral anticoagulant drug has been proven to be effective across a broad spectrum of patients in routine care (see Figure 1). However, apixaban is not for everyone. More information is needed as we shift from the era of vitamin K antagonists and enoxaparin to this new agent. The specific indications, reversal strategies, drug interactions, extremes of patient weight, bleeding, approaches to treatment failure, and use in combination with other antiplatelet drugs all require further research.
